# The assessment clock: A model to prioritize the principles of the utility of assessment formula in emergency situations, such as the COVID-19 pandemic

**DOI:** 10.15694/mep.2020.000086.1

**Published:** 2020-05-01

**Authors:** Majed Wadi, Mohamed Elhassan Abdalla, Husameldin Khalafalla, Mohamed H. Taha

**Affiliations:** 1 Medical Education Department; 2 Medical Education Centre and College of Medicine; 3 Department of Family and Community Medicine

**Keywords:** Assessment clock, assessment utility Assessment formula, e-Assessment, Remote Assessment

## Abstract

This article was migrated. The article was marked as recommended.

Many concerns have been raised regarding the impact of the changes to medical education as a result of the coronavirus disease 2019 (COVID-19) pandemic, particularly the impact of these changes on student assessments. This paper suggests an assessment clock as a conceptual model to enable medical educators to decide which assessment method is suitable under challenging circumstances, such as the COVID-19 pandemic. The assessment clock has five numbers, representing the five principles of the utility of assessment formula, which are arranged from the principle with the lowest weight (cost = 1) to the principle with the highest weight (validity = 5). The numbers are repeated in each half of the clock, and the clock is placed in the middle of two overlapping axes. The vertical axis is related to exam stakes (high or low). The low stakes condition, which represents the normal situation of running assessments at the beginning of each academic year, is placed at the top of the clock. The horizontal axis is related to the type of situation (normal or crisis). The high stakes condition is placed at the bottom of the clock. The right half of the clock represents the normal situation of planning and conducting assessments, while the left half represents an emergency situation, such as the current COVID-19 pandemic. The assessment clock offers assessment planners insights into how to determine the most important assessment principles on which they should focus during a crisis situation. Moreover, it provides practical guidance for educators to help them decide which assessment tool is suitable for use in which situation.

## Introduction

The rapid change of the coronavirus disease 2019 (COVID-19) from outbreak to pandemic has had significant consequences on education sectors worldwide, including medical education. Country-specific protective measures, such as social distancing, which aim to stop the spread of the disease, have led to medical school responses ranging from the total cessation of formal teaching and learning activities to the use of online/distance learning approaches ( Taha *et al.*, 2020).

Many concerns have been raised regarding the impact of the changes to medical education as a result of the COVID-19 pandemic, particularly the impact of these changes on student assessments ( Ahmed, Allaf and Elghazaly, 2020; Rose, 2020). The greatest worries are about the assessment of clinical competencies during end-of-year exam, and some voices are calling for the immediate graduation of final year medical students to allow them to join the healthcare workforce ( Ahmed, Allaf and Elghazaly, 2020; McVeigh, 2020). Therefore, the current dilemma for medical teachers and assessors is whether assessments should ensure the achievement of learning outcomes and the acquisition of the competencies required for safe practice ( Van Der Vleuten, 1996; Wojtczak, 2000; Cutrer *et al.*, 2017) or whether assessments should be adjusted based on extenuating circumstances, such as the social distancing measures and the needs for healthcare workforce to combat the COVID-19 pandemic. The following question has arisen in this situation: to what extent do we need to adhere to quality assessment criteria in the presence of the COVID-19 pandemic challenges?

This paper suggests the use of an assessment clock as a conceptual model to enable medical educators to decide which assessment method is suitable in difficult circumstances, such as the COVID-19 pandemic.

## The Assessment Clock Model

**Figure 1.**
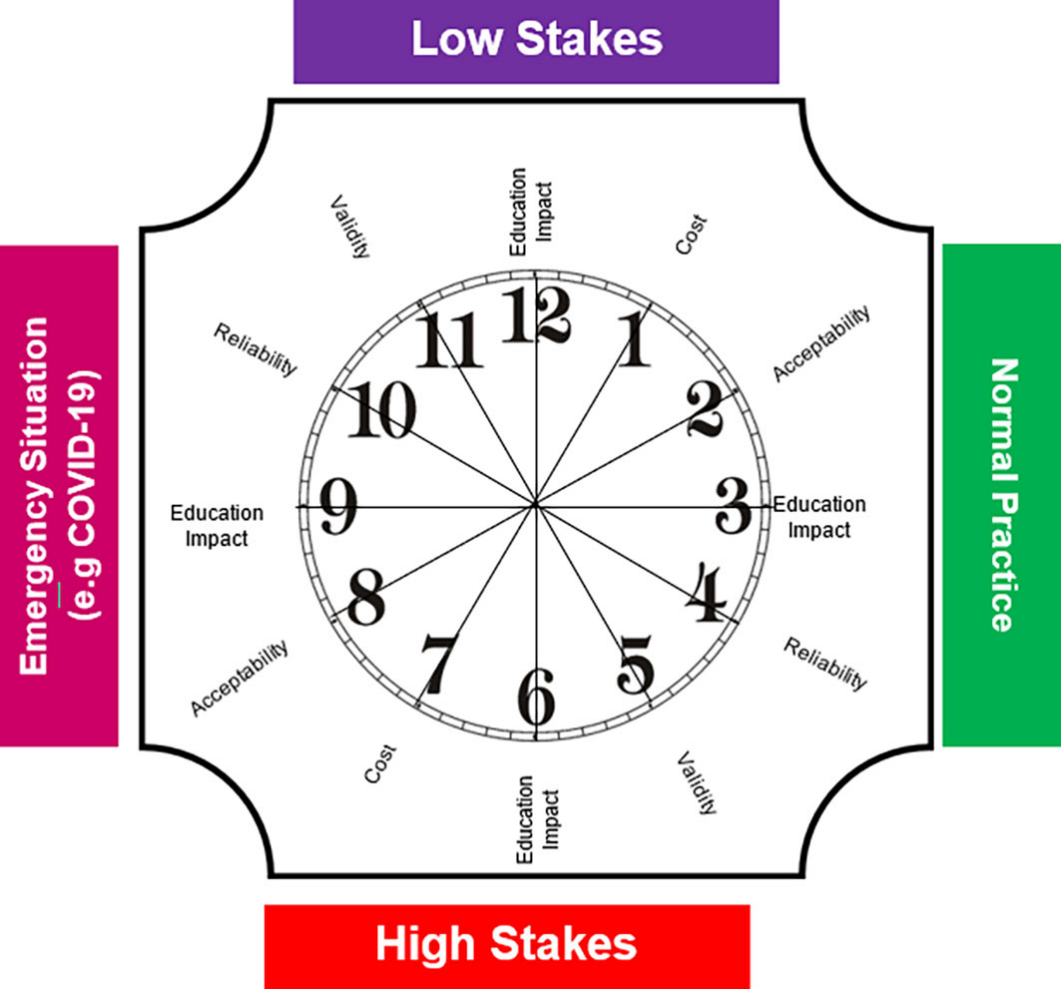
The assessment clock

The assessment clock model was developed by the authors-who work in medical education units/centres in three different universities-based on the utility of assessment (UA) formula suggested by Van Der Vleuten ( Van Der Vleuten, 1996), which incorporates five assessment principles: validity (V), which indicates the meaningfulness of the assessment tools, reliability (R), which indicates the reproducibility of the assessment tools and whether they will yield the same results; educational impact (E), which indicates the assessment’s effect on the learning process; acceptability (A), which indicates whether the assessment should be accepted by examiners and examinees; and cost or feasibility (C), which indicates whether the assessment tools are cost-effective. The formula is UA = V*R*E*A*C. The main principle of this formula involves combining different weightages, which are above zero in their sum The essential meaning of the weightages in this formula is to direct the assessor to pay more attention to the higher weighted principles and apply the best practices to achieve each principle in order to optimise the assessment practice. Usually, assessment developers maximize the weight of validity, reliability and educational impact ( Norcini and McKinley, 2007). However, the assessment clock ( Figure 1) is designed to help prioritize the adoption of the assessment criteria in different circumstances.

The model is composed of a clock with placing the five principles of assessment utility in corresponding to o’clock number. They are arranged from the principle with the lowest weight (cost = 1) to the principle with the highest weight (validity = 5). The principles are repeated in each half of the clock, with cost appearing at one and seven o’clock, and validity appearing at 5 and 11 o’clock. Since the principle of education impact is important, it is fixed at the position for each situation (3, 6, and 9 and 12 o’clock). The clock is placed in the middle of two overlapping axes. The vertical axis is related to exam stakes (high or low). The horizontal axis is related to the type of situation (normal or crisis).

The low stakes condition, which represents the normal situation of initiating assessments at the beginning of each academic year, is placed at the top of the clock. The high stakes condition is placed at the bottom of the clock. High stakes assessments are usually held at the end of each academic year. The right half of the clock represents the normal situation of planning and conducting assessments, while the left half represents an emergency situation, such as the current COVID-19 pandemic, and the obligation to perform distance learning and assessment.

Developed by the authors

## Using the Assessment Clock

The clock can be used using in two different kinds of scenarios, as discussed below.

• **The first scenario (normal practice)**

Medical schools are bound to an academic calendar, which includes many teaching and assessment activities, and assessments are assigned fixed dates in that calendar. When developing a low stakes exam, such as the formative assessment, which is usually done through e-learning platforms ( Gikandi, Morrow and Davis, 2011), attention is mainly paid to the cost, acceptability and educational impact. Therefore, the developer would subjectively consider the numerical values at the one, two and three o’clock positions, respectively, which would be reflecting their relative importance. Meanwhile, for high stakes exams, validity and reliability are more important, so the developer would pay more attention to the values at the four and five o’clock positions, respectively.

• **The second scenario (emergency situation, e.g. the COVID-19 pandemic)**

Many schools are struggling to make decisions on how to conduct high stakes exams remotely in the emergency situation created by the COVID-19 pandemic. First, attention of school leaders was on the acceptability of the tools that will be used by the assessors and the learners and cost issues (eight and seven o’clock, respectively). Regarding acceptability by students, it is an essential matter especially as there is a sudden shift to online summative eAssessment for the first time. So, it needs familiarization and explanation to students. Considering cost, conducting online assessment are costly because consideration of fidelity and security issue ( Dennick, Wilkinson and Purcell, 2009).

In other words, they were concerned with selecting the most suitable and most cost-effective online assessment tool, which would provide a fair environment for all students. Validity and reliability (10 and 11 o’clock, respectively), which are the most important principles for high stakes assessments, should not be neglected in an emergency situation. These principles could be maintained by using high-quality items from existing question banks at colleges.

For example, regarding assessing clinical competencies, only the cognitive parts of clinical competencies-such as clinical reasoning and communication skills-could be assessed during this period. Some colleges have used an objective structured video exam (OSVE). Studies have shown that OSVE is efficient, quick to administer and reliable and have demonstrated some evidence of its validity ( Humphris and Kaney, 2000).

## Conclusion

In the current COVID-19 pandemic situation, medical schools worldwide have been required to make major changes in assessments, e.g. conducting remote assessments. This shift represents a real challenge for medical educators, who must prioritize assessment criteria based on a conceptual framework in order to choose the most suitable assessment tools and instruments. The assessment clock model can help in applying that conceptual framework.

## Take Home Messages

In an emergency situation in which remote assessments must be conducted, assessment designers should use the assessment clock model to:

• evaluate the resources available to conduct remote assessments;
• focus on acceptability and feasibility, which are the first principles considered during a sudden transformation; and
• .remember to consider validity and reliability.

## Notes On Contributors

**Majed Mohamed Saleh Wadi** is a senior lecturer of Medical Education, College of Medicine, University of Qassim, KSA. He is the Head of Medical Education Department. Head of the Assessment Committee. ORCID iD: https://orcid.org/0000-0002-8117-770X.

**Mohamed Elhassan Abdalla** is an Assistant Professor of Medical Education at the College of Medicine and Medical Education Centre, University of Sharjah, UAE. He is the director of Medical Education Centre, chair of the curriculum committee. ORCID iD: https://orcid.org/0000-0002-9241-1370.

**Husameldin Khalafalla** is an Assistant Professor of Community Medicine and Medical Education at the College of Medicine, University of Jazan, KSA. ORCID iD: https://orcid.org/0000-0002-7643-6180.

**Mohamed Hassan Taha** is a visiting Assistant Professor of Medical Education at the College of Medicine and Medical Education Centre, University of Sharjah, UAE. He is the head of the Faculty Development Committee. ORCID iD: https://orcid.org/0000-0003-0808-5590.
