# Supplementary material for: The assessment clock: A model to prioritize the principles of the utility of assessment formula in emergency situations, such as the COVID-19 pandemic
Source: MedEdPublish (2016). 2020 May 1;9:86. [Version 1] doi: 10.15694/mep.2020.000086.1 (PMC10697583; doi:10.15694/mep.2020.000086.1)
Supplement: Peer review history

# Reviewer report: Reviewer response for version 1

**DOI:** 10.21956/mep.20070.r30835

**Published:** 2020-08-02

**Authors:** Hosam Eldeen Gasmalla

**Affiliations:** 1 Sudan International University

This review has been migrated. The reviewer awarded 4 stars out of 5 This interesting article addresses the dilemma of the assessment during the COVID-19 pandemic, the developed "clock" helps in the planning of the assessment, however, it would be an added value to mention more examples.

Reviewer Expertise:

NA

No decision status is available

# Reviewer report: Reviewer response for version 1

**DOI:** 10.21956/mep.20070.r30831

**Published:** 2020-05-11

**Authors:** P Ravi Shankar

**Affiliations:** 1 American International Medical University

This review has been migrated. The reviewer awarded 3 stars out of 5 This is an interesting article describing the use of an assessment clock to prioritize the utility of principles of assessment formula in emergency situations like the current pandemic. Their diagram of and description of the clock is clear. The authors mention that the clock can be used for both low stakes and high stakes exams and in normal practice and in emergency situations. However, I am still not able to fully understand how the assessment clock can be used and the authors can clarify this using more concrete examples. This will significantly strengthen the paper.

Reviewer Expertise:

NA

No decision status is available

# Reviewer report: Reviewer response for version 1

**DOI:** 10.21956/mep.20070.r30834

**Published:** 2020-05-03

**Authors:** PATRICIA CURY

**Affiliations:** 1 FACERES

This review has been migrated. The reviewer awarded 3 stars out of 5 this is a very interesting paper, but I also got lost with the clock model. It woud be very helpfull if the authors give some examples to explain better their ideas!

Reviewer Expertise:

NA

No decision status is available

# Reviewer report: Reviewer response for version 1

**DOI:** 10.21956/mep.20070.r30833

**Published:** 2020-05-03

**Authors:** Trevor Gibbs

**Affiliations:** 1 AMEE

This review has been migrated. The reviewer awarded 3 stars out of 5 I am not sure that I fully understood this paper, perhaps because when using the van der Vleuten VA formula, I always thought that many of the assessment qualities were very context and diversity driven, hence I was not sure that the authors' assessment clock was not that universally acceptable. Plus I feel that within the Covid-19 pandemic, very little is universally acceptable and all of the VA qualities are justifiable. It would have been very helpful had the authors explored this and given more concrete examples to justify their points.

Reviewer Expertise:

NA

No decision status is available

# Reviewer report: Reviewer response for version 1

**DOI:** 10.21956/mep.20070.r30830

**Published:** 2020-05-02

**Authors:** Hui Meng Er

**Affiliations:** 1 International Medical University

This review has been migrated. The reviewer awarded 4 stars out of 5 The dilemma faced by medical educators around the world in conducting student assessments during the Covid-19 pandemic are well described by the authors. Given the common challenges encountered by most institutions in this unprecedented crisis situation, shared guidelines and practices among academic institutions are useful, however, should be further contextualised to the institutional needs and resources. The conceptual model of an assessment clock that is founded on the principles of assessment utility is practical, realistic and serves as a guide for rationalising assessment tools and platform during an emergency situation. In addition to acceptability by the examiners and learners, the acceptability of the alternative assessment tools by the accreditation and professional bodies should also be considered.

Reviewer Expertise:

NA

No decision status is available

# Reviewer report: Reviewer response for version 1

**DOI:** 10.21956/mep.20070.r30832

**Published:** 2020-05-01

**Authors:** Felix Silwimba

**Affiliations:** 1 University of Lusaka

This review has been migrated. The reviewer awarded 5 stars out of 5 this article is informative and challenging to medical educators .

Reviewer Expertise:

NA

No decision status is available
